# Management of acute ischemic stroke

**DOI:** 10.4103/0974-2700.66552

**Published:** 2010

**Authors:** Vishal Sharma, Alka Sharma

**Affiliations:** Department of Medicine, University College of Medical Sciences, Delhi, India

Sir,

We read the otherwise brilliant review on emergency care for cerebrovascular accidents.[1] We have few observations regarding the management of acute ischemic stroke.

Recently, the recommendation regarding administration of tissue plasminogen activator in acute ischemic stroke has been modified and has been increased from 3 to 4.5 h on the basis of the findings of the third European Cooperative Acute Stroke Study (ECASS-3).[2] While the review correctly recommends the use of computed tomography as the initial rapid study, it is important to realise the importance of magnetic resonance imaging (MRI) in the diagnosis of acute ischemia as also excluding mimics. MRI has not only become more readily available but is also quicker to perform than before. The ability of diffusion-weighted imaging to identify acute infarction and of fluid-attenuated inversion recovery sequence and gradient echo in detecting hemorrhage argue for a greater role of MRI in acute stroke. Recent recommendations have indicated that MRI can be utilized in the first 3 h if it does not delay administration of thrombolytic therapy.[3] Also, the review does not mention the need to control hyperglycemia in acute ischemic stroke, which may have some benefit in improving the outcome.[4]
